# Impacts of early postpartum behavioral patterns on the fertility and milk production of tropical dairy cows

**DOI:** 10.14202/vetworld.2025.1109-1126

**Published:** 2025-05-13

**Authors:** Aqeel Raza, Kumail Abbas, Theerawat Swangchan-Uthai, Henk Hogeveen, Chaidate Inchaisri

**Affiliations:** 1 International Graduate Program of Veterinary Science and Technology, Faculty of Veterinary Science, Chulalongkorn University, Bangkok, 10440, Thailand; 2 Research Unit of Data Innovation for Livestock, Department of Veterinary Medicine, Faculty of Veterinary Science, Chulalongkorn University, Bangkok, 10330, Thailand; 3 Center of Excellence in Animal Fertility Chulalongkorn University (CU-AF), Department of Obstetrics, Gynecology, and Reproduction, Faculty of Veterinary Science, Chulalongkorn University, Bangkok, 10330, Thailand; 4 Business Economic Group, Wageningen University and Research, Wageningen, 6706KN Wageningen, The Netherlands

**Keywords:** K-means clustering, milk yield, parity, postpartum behavior, reproductive performance, smart sensors, tropical dairy cattle

## Abstract

**Background and Aim:**

Early postpartum behavioral patterns are pivotal indicators of dairy cow health, reproductive success, and lactation performance, particularly under the environmental stressors of tropical climates. This study aimed to investigate how these behavioral patterns, as captured by smart biosensor data, influence reproductive outcomes, and milk yield in Holstein Friesian cows, with specific emphasis on parity differences and behavioral clustering.

**Materials and Methods:**

A total of 227 Holstein Friesian cows, categorized by parity (primiparous vs. multiparous), were monitored using AfiTag-II accelerometers from 3 days prepartum to 30 days postpartum. Behavioral variables – activity, rest time, rest per bout, and restlessness ratio – were subjected to K-means clustering to identify distinct behavioral profiles. Reproductive performance was analyzed using Cox proportional hazard models, while lactation dynamics were modeled using the Wood function to estimate peak yield, peak time, and persistency.

**Results:**

Three distinct behavioral clusters were identified. Primiparous cows in Cluster 1 showed the highest early postpartum activity (~300 min/day at 5 days in milk [DIM]) and restlessness ratios, while multiparous cows exhibited more stable behavioral profiles. Cox regression suggested that cows in Cluster 0 had a higher, although non-significant, likelihood of estrus onset at 40 DIM (Hazard ratio = 1.44, p = 0.09). Lactation modeling revealed that multiparous cows in Cluster 0 attained the highest cumulative milk yield (4896.6 ± 252.1 kg at 305 DIM), while the single cow in Cluster 2 exhibited an atypical lactation curve with a delayed peak and reduced persistency.

**Conclusion:**

Postpartum behavioral clustering reveals parity-specific lactation and reproductive trajectories in tropical dairy cows. Higher activity and restlessness ratios may delay estrus and compromise milk yield, underscoring the potential of behavioral monitoring for targeted reproductive and nutritional management. Integration of sensor-based clustering with routine herd monitoring may support early identification of cows at risk of suboptimal performance, improving reproductive efficiency and milk production in tropical dairy systems.

## INTRODUCTION

Postpartum behavioral patterns are vital indicators of dairy cow welfare and exert a direct influence on reproductive efficiency and milk production outcomes [1, 2]. Traditional methods for classifying cow behavior rely on manual observation and predefined criteria [3, 4]; however, these approaches are labor-intensive, time-consuming, and prone to observer bias [5]. Recent advancements in automated, sensor-based technologies – such as the AfiTag-II biosensor – now provide robust tools for continuous monitoring of activity and resting behavior in dairy cows. These devices facilitate precise, high-frequency data acquisition, enabling a reliable assessment of the associations between postpartum behavioral changes and reproductive and productive performance [2, 6]. In addition to minimizing labor demands, these automated systems enhance herd management efficiency and contribute to improved economic outcomes on dairy farms [7, 8].

Behavioral patterns in dairy cows differ markedly between tropical and temperate climates, largely due to environmental stressors such as high ambient temperatures and humidity [9, 10]. In tropical regions, these stressors often result in irregular resting behavior and delayed reproductive cycles, in contrast to the more predictable patterns observed in temperate zones [11]. Effectively addressing these climate-induced differences necessitates management strategies tailored to local environmental conditions, thereby optimizing fertility and milk production in tropical herds [12]. Given the critical economic importance of reproductive success, early postpartum estrus detection and efficient insemination are essential for maintaining productivity in dairy operations [13]. However, heat stress in tropical climates frequently alters postural and activity-related behaviors, which may negatively affect key indicators of reproductive performance and productivity.

The lactation curve (LC) serves as a valuable analytical tool for understanding milk production dynamics, particularly in tropical environments where atypical LC patterns are more frequently observed due to environmental stressors [14–16]. Metrics such as peak time, peak yield, and persistency are instrumental in evaluating production potential and overall health, with these parameters influenced by factors including parity, genetic background, and environmental conditions [17, 18]. In tropical climates, approximately 20%–30% of dairy cows may exhibit atypical LC shapes – such as delayed peak production or reduced persistency – stemming from elevated metabolic stress during early lactation [14, 19, 20]. Insight into these variations is essential for designing management practices that support both productivity and animal welfare in tropical dairy systems.

Clustering analysis has emerged as a valuable technique for classifying cows based on behavioral and physiological profiles. In particular, the K-means clustering algorithm is well-suited for analyzing large datasets and identifying distinct behavioral patterns, especially under the variable conditions’ characteristic of tropical environments [15, 21]. While previous research by Rebuli *et al*. [22], Grelet *et al*. [23], and Pereira *et al*. [24] have applied K-means clustering to classify cows based on milk yield, genetic traits, and blood parameters, relatively few studies have employed this method to forecast reproductive performance and LC characteristics in tropical dairy populations.

In this study, we employed K-means clustering to categorize activity-related behavioral patterns during the early lactation period in tropical dairy cows, with specific attention to parity-based differences. By integrating behavioral clustering with LC modeling using the Wood function [17], our objective was to predict reproductive and lactation outcomes associated with specific behavioral profiles. This approach aims to inform the development of targeted management strategies for tropical dairy production systems.

Despite growing interest in the application of sensor-based monitoring systems for dairy cow behavior, limited research has investigated the integration of early postpartum behavioral data with reproductive and lactation outcomes under tropical environmental conditions. Most existing studies have focused on temperate climates, where environmental stressors differ significantly from those encountered in tropical regions. Moreover, while K-means clustering has been employed to classify cows based on milk yield, genetic traits, or metabolic profiles, its application to behavior-based classification during the critical transition period remains underexplored in tropical dairy systems. Notably, the predictive relationship between cluster-defined behavioral profiles and reproductive milestones such as estrus onset, as well as lactation dynamics modeled through established functions like the Wood model [17], has not been sufficiently characterized. This represents a key research gap with practical implications for optimizing herd fertility and milk production strategies in heat-stressed environments.

Accordingly, the present study aimed to evaluate the effects of early postpartum behavioral patterns on reproductive performance and milk yield in tropical Holstein Friesian cows, using K-means clustering to identify distinct behavior profiles. These behavioral clusters were subsequently analyzed in relation to reproductive traits using Cox regression and lactation dynamics through the Wood function model [17]. The study also examined parity-specific differences in behavioral and production responses, thereby contributing to a data-driven framework for precision dairy management in tropical climates.

## MATERIALS AND METHODS

### Ethical approval and informed consent

This study utilized data directly retrieved from the database of a commercial dairy farm, focusing on early postpartum activity patterns, reproductive outcomes, and milk production performance. As a retrospective study based on secondary data analysis, with no direct or indirect interaction with animals, formal approval from the Institutional Animal Care and Use Committee was not required. Nonetheless, the study was conducted in accordance with ethical research standards, including data protection and confidentiality. Before data collection, written informed consent was obtained from the farm owner, who was thoroughly informed about the study’s objectives, the intended use of the data, and the measures implemented to ensure confidentiality and anonymity. All procedures adhered to relevant ethical guidelines, maintaining the privacy and protection of both the owner and the data involved.

### Study period and location

Data on calving events that occurred between July 2020 and May 2022 were collected from 227 Holstein Friesian dairy cows housed at Sithichoke Dairy Farm, located in Nakhon Ratchasima Province, Thailand.

### Animals, housing, diet, management, and health

The sample consisted of 173 first-lactation cows and 53 cows in their second or higher lactation. These cows exhibited an average lactation length of 152.50 ± 8.33 days and a gestation period of 279.47 ± 20.11 days (mean ± standard deviation). Inclusion criteria required complete activity and milking records from 3 days prepartum to 30 days postpartum for behavioral analysis, and from 0 to 305 days in milk (DIM) for milk yield data. Cows with missing identification data, incomplete lactation records, or faulty sensor readings were excluded to maintain dataset integrity.

The cows were housed in a free-stall barn aligned southwest to northeast, incorporating designated feed storage, resting, feeding, and delivery areas. A partially slatted central resting area was extended along its full length. The milking parlor was located on the southwest side of the farm, adjacent to the office and milk storage facility. Natural light was supplemented by artificial lighting managed by farm staff to support feeding and behavioral monitoring.

Thailand’s climate is marked by high ambient temperatures and humidity, with average values of 27°C and 74%, respectively [25]. Seasonal variation was observed: 20°C–26°C in winter (November–February), 32°C–37°C in summer (March–June), and 26°C–30°C during the rainy season (July–October). Relative humidity ranged from 65% to 70% in winter, 75% to 80% in summer, and often exceeded 80% in the rainy season [25–27]. To mitigate heat stress, cooling strategies such as shaded resting areas, sprinklers, and ventilation fans were implemented [10].

Milking occurred twice daily, from 05:30 to 06:00 and from 16:30 to 17:00. Cows were grouped by production level and fed a total mixed ration twice daily (06:00–06:30 and 17:00–17:30), formulated to meet their nutritional needs. Daily feed intake was monitored by weighing the feed provided. The chemical composition of the ration is presented in Table 1. Clean water was available ad libitum, and all animals were under the supervision of zootechnical and veterinary staff throughout the study.

**Table 1:** Description of chemical composition of feed ingredients used in this study.

Chemicals	TMR 1	TMR 2	TMR 3	TMR 4
Proximate analysis on a dry matter				
Dry matter (%)	26.92	37.62	32.62	36.66
Crude protein (%)	10.53	13.82	14.28	16.66
Crude fat content (%)	1.70	1.39	1.40	2.03
Crude fiber (%)	20.89	20.93	24.00	27.45
NFE (Mcal/kg)	52.44	35.19	33.05	27.72
Detergent analysis on a dry matter basis (%)				
Ash	14.44	28.67	27.27	26.14
ADF	43.00	26.26	28.75	25.03
NDF	67.21	36.55	43.77	39.26
ADL	4.32	2.94	3.42	3.29
Cellulose	38.68	23.32	25.33	21.74
Hemicellulose	24.21	10.29	15.02	14.23

NFE=Energy content (Mcal/kg), ADF=Acid detergent fiber, NDF=Neutral detergent fiber, ADL=Acid detergent lignin, TMR=Total mixed ration. TMR 1=Dry cow group, TMR 2=Low milk production group: <10 kg/day, TMR 3=Medium milk production group: ≥10–25 kg/day, TMR 4=High milk production group: >25 kg/day.

A secondary dataset containing the same 227 animals was used to assess behavioral variables – activity, rest time, rest per bout, and restlessness ratio – collected from −3 to 30 DIM. A 7-day rolling average was applied to smooth these variables for analysis.

Farm staff conducted daily health assessments to monitor for signs of illness, injury, or behavioral abnormalities. Routine veterinary inspections were performed twice daily following feeding. Preventive health measures, including vaccinations and parasite control programs, were in place to ensure herd health. All health records were systematically maintained in the farm’s database for reference and analysis.

Transition cows were managed according to standard farm protocols, which included relocation to calving pens based on prior insemination and health history. The calving barn, equipped with rubber mattresses, was located adjacent to the lactating cow barn and within visual proximity. Bedding was cleaned and replaced after each calving. Additional information on calving protocols is available in our previous study by Raza *et al*. [10].

### Data preparation

#### Sensor-based data preprocessing

The AfiTag-II biosensor (Afikim Ltd., Kibbutz, Israel) is a proprietary tri-axial accelerometer encased in a durable housing, designed to capture X-, Y-, and Z-axis movement data. The sensor was securely fastened to the right hind leg of each cow, approximately 20 cm above the hoof, using adjustable straps to ensure comfort and freedom of movement. The device converts mechanical acceleration – such as movement and gravitational force – into waveform signals [10, 28], enabling the detection and classification of both static and dynamic activities [29]. Behavioral metrics recorded included activity, rest time, rest per bout, and restlessness ratio, as summarized in Table 2.

**Table 2:** Definition of postural behavioral metrics.

Parameters	Definition
Activity	Total duration of physical movement of the animal per day.
Rest time	The total duration of resting period throughout the day.
Rest periods per Bout	Average resting period of individual cow rest.
Restlessness ratio	A metric designed to quantify a cow’s relative activity by comparing its current activity level to its individual baseline.

Activity and rest time were measured in minutes per day; rest per bout was measured in minutes per bout per day. The restlessness ratio was calculated as the ratio of the current day’s activity time (in minutes) to the average activity time of the previous 7 days.

Sensors were installed approximately 1 month before calving and remained in place throughout the study. All devices were synchronized with the farm’s milking software to link behavioral data with individual animal identification. Cows were provided a 1-week adaptation period. Previous studies by Raza *et al*. [10], Papageorgiou *et al*. [30], and Henriksen and Munksgaard [31] have validated the accuracy of the AfiTag-II system in detecting behavioral patterns. Calibration procedures accounted for leg angle, weight distribution, and movement variability to ensure reliable measurements. Any sensor exhibiting malfunction was recalibrated or replaced before final data collection.

To ensure data quality, the research team implemented strict protocols to address potential sources of measurement bias. Factors such as gait pattern variation, body condition score, and leg strap tightness were monitored. Sensors were attached by trained personnel from the device manufacturer, following a standardized protocol for placement. Farm staff also periodically inspected sensor positioning to ensure uniform application across all animals.

Behavioral data were analyzed using inbuilt algorithms and recorded in units of minutes per day. Data were transmitted wirelessly to a base station and subsequently downloaded to Microsoft Excel 2021 [32]. The activity dataset (n = 227) contained no missing values. Minor gaps (~2%) in the milk yield dataset were addressed using the Wood lactation model and linear interpolation. Outliers were identified using the interquartile range (IQR) method in the Statistical Package for the Social Sciences (SPSS) version 29.0.1 (IBM Corp., NY, USA). To reduce skewness, data normalization was performed using Z-score and log transformation in Python (version 3.12.2, Python Software Foundation, https://www.python.org). The complete preprocessing workflow and scripts are publicly available through GitHub: https://github.com/AqeelRaza51214.

#### Clustering

Clustering is a foundational data mining and machine learning approach used to group similar data points into distinct, non-overlapping clusters [33]. A core component of clustering is the quantification of similarity or distance between data points [34]. The Euclidean distance is commonly applied to measure the straight-line distance in multidimensional datasets [35]. Within clustering applications, this metric helps determine the proximity of individual data points to their respective cluster centroids [36]. The mathematical representation of the Euclidean distance “d” is given as follows:


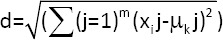


Where:

a) x_ij_ indicates the value j-th recorded feature for cow, i.
b) μ_kj_ is the value of the centroid for j-th recorded features in cluster k.
c) m is the number of recorded features that were used in the analysis.

To evaluate the effectiveness of the clustering outcome, it is necessary to assess both cluster cohesion and separation. This study employed the Within-Cluster Sum of Squares (WCSS) method to quantify total variance within each cluster based on selected features. WCSS was computed by summing the squared Euclidean distances between individual data points and the centroid of their respective clusters. The corresponding mathematical equation is shown below.


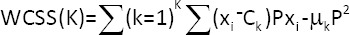


Where:

a) x_i_ denotes the recorded features cow i.
b) c_k_ stands for the number of cows assigned to cluster k.
c) μ_k_ is the centroid of cluster k, representing the mean values of recorded features variables for that cluster.
d) ║x_i_=μ_k_║^2^ is squared Euclidean distance between the recoded features vector x_i_ of cow i and the cluster centroid μ_k_.
e) The outer summation: 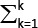 iterate over each k cluster.
f) The inner summation: 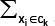 shows the sum of square distances for all cows assigned to cluster k.

**Supplementary Figure 1:**
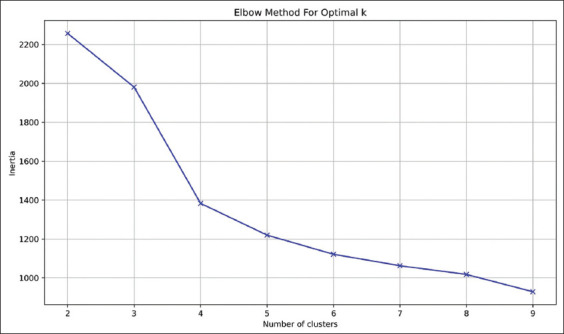
Elbow method for identification of clusters numbers.

**Supplementary Figure 2:**
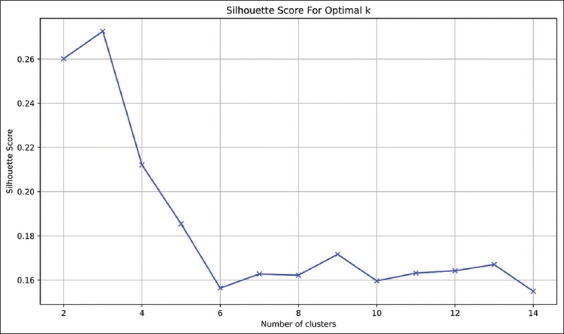
Silhouette score for detection of clusters numbers.

K-means clustering, an unsupervised machine learning algorithm, was utilized due to its efficiency with both simple and high-dimensional datasets. However, K-means is known to be sensitive to outliers, as extreme values can distort the cluster centroids and overall data distribution [14, 34]. To enhance clustering validity and determine the optimal number of clusters, three complementary methods were applied: the elbow method, the silhouette score index, and WCSS. The elbow method is a visual tool that plots the WCSS against varying cluster counts to identify the point where additional clusters provide diminishing improvements in cohesion [14, 37]. The silhouette score quantifies cluster separation by comparing the mean intra-cluster similarity with the nearest-cluster similarity [38]. Based on these techniques, three clusters were identified as optimal for the present dataset (Supplementary Figures 1 and 2). Extensive data preprocessing was performed to minimize the influence of outliers, including the IQR method for outlier detection, and Z-score and log transformation for data normalization and skewness reduction.

#### Characterization and comparison of clusters

To determine distinct behavioral clusters within the second dataset, K-means clustering was applied using the following postural behavior variables: activity, rest time, rest per bout, restlessness ratio, parity (Lactation No. 1 vs. Lactation No. ≥2), DIM (−3 to 30 days), and their corresponding 7-day rolling averages (rolling activity, rolling rest time, rolling rest per bout, and rolling restlessness ratio). The resulting cluster classifications were merged with the milk yield dataset to enable a comprehensive comparison of lactation characteristics by parity group. Specifically, comparisons were made between primiparous and multiparous cows for peak milk yield, peak time (DIM of peak yield), and cumulative milk yield at 56, 84, 126, and 305 DIM. The LCs for each cluster were modeled using the Wood function [17], incorporating cluster, parity, and milk yield data to explore associations between postural behavior and lactation performance.

#### Milk dataset

The milk dataset was obtained from a commercial dairy farm equipped with the AfiMilk automatic milking system (Afikim Ltd.), operational between December 2019 and July 2023. Milk yield data were recorded for individual cows using radio-frequency identification tags integrated into the system. The dataset included 227 lactation records, each representing a unique cow. Records comprised milking date, cow ID, parity (first lactation or second and above), DIM (0–305 DIM), and daily milk yield in kilograms.

Missing values in the milk yield data were imputed using the Wood lactation model [17], as peak yield and persistency are essential for LC analysis. The model, based on a gamma function, assumes a biologically plausible lactation trajectory: An initial increase in milk production post-calving, followed by a peak and gradual decline. While the model does not account for external variables such as disease, feed variation, seasonality, or heat stress, it remains a preferred choice due to its simplicity, robustness in trend analysis, and suitability for imputing missing data. Each cow’s LC was fitted using the Wood model, and the resulting parameters were used to estimate missing daily yield values. Peak yield and other milk production traits were also calculated using this model. The Wood model is expressed mathematically as follows:

y = at^b^ e^-ct^

Where:

a) “y” represents the daily milk yield.
b) “t” is the time since parturition.
c) “a” denotes the overall milk production levels.
d) “b” signifies the ascending phase of lactation that leads to the peak yield.
e) “c” demonstrates the descending phase of milk yield after parturition.

To validate the model, two metrics were used: The coefficient of determination (R^2^) and mean absolute error (MAE). R^2^ quantifies how well the model explains the variance in milk yield, while MAE reflects the average magnitude of prediction error. Tables 3–5 [17, 39] summarize lactation performance values and the respective validation metrics, highlighting model performance in relation to parity.

**Table 3:** Descriptive statistics of milk yield parameters in primiparous cows (n = 173).

Variables	Primiparous				Cluster (0)				Cluster (1)				Cluster (2)	
			
	n	Mean ± SE	Minimum	Maximum	n	Mean ± SE	Minimum	Maximum	n	Mean ± SE	Minimum	Maximum	n	Absolute value
Peak time	174	52.28 ± 2.06	0.00	204.08	59	50.82 ± 3.45	0.00	113.68	114	52.92 ± 2.59	0.00	204.8	1	68.82
Peak yield	174	18.74 ± 0.25	12.24	29.17	59	18.95 ± 0.43	13.29	28.73	114	18.68 ± 0.30	12.24	29.17	1	13.31
Persistency	174	54.40 ± 1.05	10.97	99.37	59	52.04 ± 1.68	24.38	85.95	114	55.64 ± 1.33	10.97	99.37	1	52.02
MY 56 days	174	941.22 ± 14.51	473.92	1574.24	59	944.79 ± 25.31	573.93	1526.99	144	942.18 ± 17.71	473.92	1574.24	1	621.30
MY 84 days	174	1449.62 ± 20.51	851.67	2329.42	59	1456.06 ± 35.06	978.76	2225.74	114	1450.29 ± 25.31	851.67	2329.42	1	993.21
MY 126 days	174	2172.94 ± 28.77	1370.33	3378.78	59	2181.86 ± 48.72	1536.20	3158.45	114	2173.94 ± 35.65	1370.33	3378.79	1	1532.17
My 305 days	174	4530.08 ± 60.48	3100.76	6820.40	59	4512.76 ± 104.95	3100.76	6806.45	114	4550.29 ± 7.09	3185.19	6820.40	1	3247.15

Peak time: Number of days in milk (DIM) at which peak was predicted. Peak Yield: Peak milk yield (kg/day). Persistency: Describes the ability of the cow to maintain a relatively stable milk yield after reaching peak production, expressed as the rate of decline in milk yield over time Burgers *et al*. [39]. MY=Milk yield (kg/day), SE=Standard error. The predicted values of variables are based on the Wood model equation Wood [17]; Peak time: b/c; b=rate of increase in milk yield leading up to peak; c=rate of decline in milk yield after peak; Peak yield: 
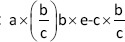
, Persistency: 100 × (milk at 305 DIM)/(Peak yield). Cluster 2: Contains only single animal; the SE was not calculated.

**Table 4:** Descriptive statistics of milk yield parameters in multiparous cows (n = 53).

Variables	Multiparous				Cluster (0)				Cluster (1)			
		
	n	Mean ± SE	Minimum	Maximum	n	Mean ± SE	Minimum	Maximum	n	Mean ± SE	Minimum	Maximum
Peak time	53	51.39 ± 3.11	0.00	105.42	7	47.45 ± 13.91	0.00	94.71	46	51.99 ± 2.97	0.00	105.42
Peak yield	53	20.33 ± 0.44	12.65	26.82	7	20.92 ± 1.06	18.14	26.64	46	20.24 ± 0.48	12.65	26.82
Persistency	53	51.01 ± 1.81	28.77	91.36	7	51.75 ± 4.94	33.38	72.83	46	50.90 ± 1.97	28.77	91.36
MY 56 days	53	1019.84 ± 24.46	584.74	1438.54	7	1038.80 ± 83.01	780.92	1435.84	46	1016.96 ± 25.61	584.74	1438.54
MY 84 days	53	1572.47 ± 35.03	943.39	2126.27	7	1589.28 ± 105.48	1314.27	2126.27	46	1569.92 ± 37.51	943.39	2114.76
MY 126 days	53	2353.64 ± 49.73	1465.73	3078.47	7	2369.93 ± 132.47	2067.25	3078.47	46	2351.16 ± 54.17	1465.74	3030.68
MY 305 days	53	4826.97 ± 103.88	3121.47	6103.73	7	4896.57 ± 252.14	4089.90	6103.73	46	4816.38 ± 114.27	3121.47	6101.23

Peak time: Number of days in milk (DIM) at which peak was predicted. Peak Yield: Peak milk yield (kg/day). Persistency: Describes the ability of the cow to maintain a relatively stable milk yield after reaching peak production, expressed as the rate of decline in milk yield over time Burgers *et al*. [39]. MY=Milk yield (kg/day), SE=Standard error. The predicted values of variables are based on the Wood model equation Wood [17]; Peak time: b/c; b=rate of increase in milk yield leading up to peak; c=rate of decline in milk yield after peak; Peak yield: 
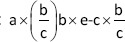
, Persistency: 100 × (Milk at 305 DIM)/(Peak yield).

**Table 5:** Validation of Wood lactation curve model for the parity and cluster groups (n = 227).

Parity	Cluster	R^2^	MAE
Primiparous	0	0.83	1.35
	1	0.69	1.05
	2	0.83	1.27
Multiparous (≥2)	0	0.80	1.48
	1	0.82	1.43

R² (coefficient of determination) measures the proportion of the observed milk yield variance explained by the model, indicating the overall fit. MAE (mean absolute error) represents the average absolute difference between predicted and observed values, reflecting typical prediction error.

#### Programming packages

Data cleaning, preprocessing, and imputation using the Wood model [17] were conducted using Python (version 3.12.2; https://www.python.org/). Key libraries included NumPy (version 1.24.4, NumPy Developers, https://numpy.org) [40, 41], and Pandas (version 2.2.3, pandas development teams, https://pandas.pydata.org) with the Wood model implementation executed through the SciPy package (version 1.14.1, https://scipy.org) [42]. All analyses were performed in Jupyter Notebook (https://jupyter.org). LC visualization was completed using Matplotlib and Seaborn libraries [43]. For reproducibility, the Python scripts, cleaned datasets, and additional clustering and LC analysis files are available at the GitHub repository: https://github.com/AqeelRaza51214.

### Statistical analysis

Descriptive statistics were generated using SPSS version 29.0.1 (IBM Corp., NY, USA). Outliers were removed using the IQR method. An exploratory analysis was performed to investigate potential predictors of reproductive milestones: calving to first heat, calving to first service, and calving to conception.

To assess reproductive performance, univariable Cox proportional hazards regression was conducted to calculate hazard ratios (HRs) for achieving first heat at 40, 60, and 90 DIM; first service at 60, 90, and 120 DIM; and pregnancy at 100, 150, and 200 DIM. Cluster 1 was designated as the reference group. Cows that achieved reproductive outcomes before the respective DIM thresholds were censored from the analysis. Adjusted HRs were reported alongside 95% confidence intervals (CI) and corresponding p-values. p ≤ 0.05 was considered statistically significant.

Lactation performance was evaluated by comparing peak milk yield, peak time, persistency, and cumulative milk yields at 56, 84, 126, and 305 DIM across clusters, within each parity group (primiparous and multiparous). Normality was assessed using the Shapiro–Wilk test and Q-Q plots. For normally distributed variables, a one-way analysis of variance (ANOVA) was used, followed by Tukey’s honestly significant difference test for *post hoc* comparisons. Non-normal variables were analyzed using the Kruskal–Wallis test, with pairwise comparisons conducted using Dunn-Bonferroni correction.

Effect sizes were reported to enhance interpretability: Partial eta squared (η^2^) for ANOVA and epsilon squared (ε^2^) for the Kruskal–Wallis test. All statistical analyses were stratified by parity to account for physiological differences. The significance level was set at p < 0.05 for all comparisons.

## RESULTS

### Temporal dynamics of postural behavior across parity and clusters

**Figure 1:**
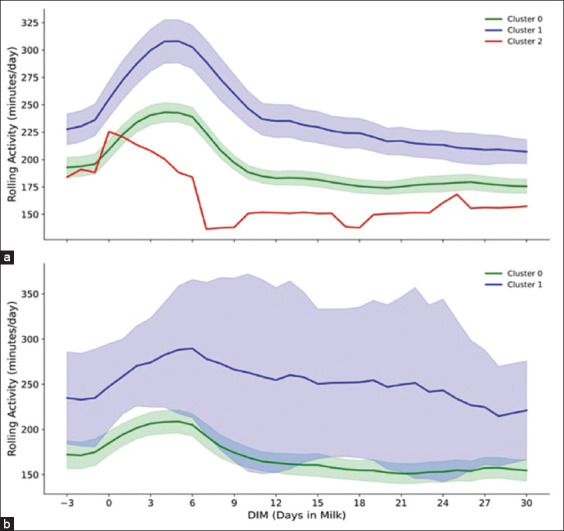
The figure illustrates (a) rolling activity (minutes/day) of primiparous cows (n = 173) across three cluster groups (0, 1, and 2) and (b) rolling activity (minutes/day) of multiparous cows (n = 53) across two cluster groups (0, and 1), with 95% confidence interval during the −3 to 30 days postpartum.

**Figure 2:**
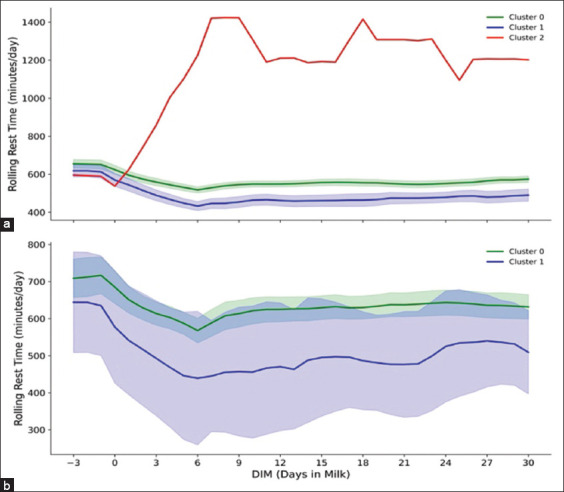
The figure display (a) rolling rest time (minutes/day) of primiparous cows (n = 173) and (b) rolling rest time (minutes/day) of multiparous cows (n = 53) in two clusters groups (0, and 1) across −3 to 30 days postpartum, with 95% confidence interval.

**Figure 3:**
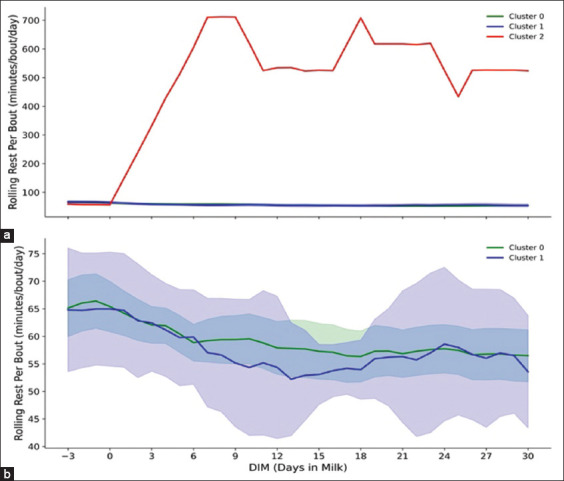
The figure explains (a) rolling rest per bout (minutes/bout/day) of primiparous cows (n = 173) across three cluster groups (0, 1, and 2) and (b) rolling rest per bout (minutes/bout/day) of multiparous cows (n = 53) across two cluster groups (0, and 1), with 95% confidence interval, across −3 to 30 days postpartum.

**Figure 4:**
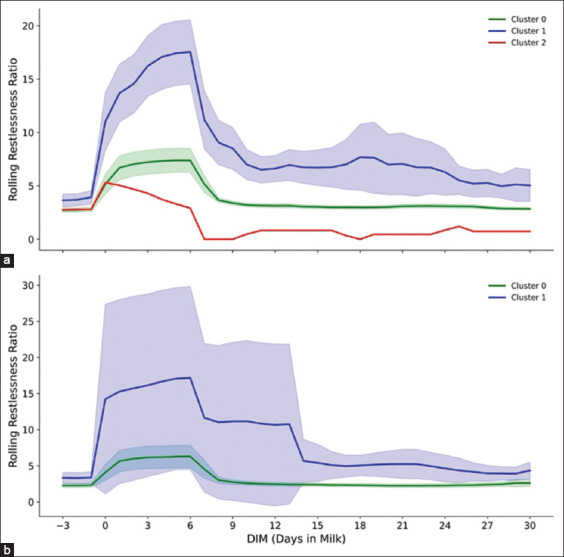
The figure demonstrates (a) rolling restlessness ratio of primiparous cows (n = 173) in three cluster groups (0, 1, and 2) and (b) rolling restlessness ratio of multiparous cows (n = 53) in two cluster groups (0, and 1) across −3 to 30 days in milk postpartum, with 95% confidence interval.

Figures 1–4 present a detailed evaluation of postural behaviors and resting patterns, illustrating rolling activity, rolling rest time, rest duration per bout, and restlessness ratio among dairy cows, stratified by parity and behavioral clusters.

For multiparous cows (Figure 3b), both clusters demonstrated a decline in rest per bout duration approaching calving – Cluster 0 averaging ~65 min and Cluster 1 slightly lower at ~60 min. Post-calving, both groups gradually recovered, though durations remained below prepartum levels. Cluster 0 cows displayed more stable rest patterns overall.

Figures 4a and b show the restlessness ratio, a behavioral indicator of agitation. Primiparous cows in Cluster 1 showed the highest restlessness, peaking at ~18 on the day of calving, then declining to ~10 by 15 DIM. Cluster 0 cows peaked at ~6 and stabilized at 4–5, while the single cow in Cluster 2 maintained a consistently low ratio of ~2–3.

Rolling activity patterns from −3 to 30 DIM revealed distinct behavioral differences among primiparous cows across clusters (Figure 1a). Cluster 1 cows exhibited the highest early postpartum activity, peaking at approximately 300 min/day shortly after calving, followed by a progressive decline. In contrast, cows in Cluster 0 maintained a more stable activity pattern, averaging 225 min/day. The single primiparous cow in Cluster 2 showed reduced and inconsistent activity levels.

A similar trend was observed in multiparous cows (Figure 1b). Cluster 1 cows showed elevated activity early postpartum, peaking at around 350 min/day near 5 DIM. However, activity levels declined thereafter. Cows in Cluster 0 maintained relatively steady activity, averaging approximately 200 min/day.

Resting time patterns, depicted in Figures 2a and b, indicated that primiparous cows in Clusters 0 and 1 exhibited reduced resting time leading up to calving (Figure 2a), with a nadir around 5 DIM. Post-calving, Cluster 0 cows maintained a higher average rolling rest time (~600 min/day) compared to Cluster 1 cows (~550 min/day). In contrast, the cow in Cluster 2 exhibited an atypical increase in resting time post-calving, reaching a peak of 1200 min/day.

For multiparous cows (Figure 2b), both Clusters 0 and 1 displayed declining rest time approaching calving. Postpartum, Cluster 0 cows stabilized around 650 min/day, whereas Cluster 1 cows initially declined to 400 min/day and gradually recovered to ~500 min/day by 30 DIM.

Figure 3a illustrates rest per bout durations in primiparous cows. Clusters 0 and 1 maintained relatively stable durations (~90 min/bout/day). The cow in Cluster 2 deviated significantly, with rest per bout increasing sharply post-calving to over 700 min/bout/day.

Among multiparous cows (Figure 4b), Cluster 1 again exhibited the highest restlessness ratio, peaking above 20 on calving day and declining to ~8 by 15 DIM. Cluster 0 cows showed a moderate peak (~6) and stabilized around 4–5 thereafter.

### Reproductive performance

The reproductive performance of 226 Holstein Friesian cows was evaluated under tropical conditions, with 76.2% (n = 173) in their first lactation and 23.4% (n = 53) in their second or subsequent lactation (≥2). Cows were categorized into two primary behavioral clusters: Cluster 0 (n = 66, 29.2%) and Cluster 1 (n = 160, 70.7%). A single cow in Cluster 2 was excluded from regression analysis due to its singular representation.

Univariable Cox regression analysis designated Cluster 1 as the reference category (Table 6). The analysis indicated a delayed return to estrus in Cluster 1. At 40 DIM, cows in Cluster 0 were 44% more likely to show estrus than those in Cluster 1 (HR = 1.44, p = 0.09). This likelihood decreased to 32% at 60 DIM (HR = 1.32, p = 0.10) and to 22% at 90 DIM (HR = 1.22, p = 0.19).

**Table 6:** Univariate Cox regression analysis of reproductive variables.

Variables	Cluster	n	Β	SE	p-value	HR	95% CI	

							Lower	Upper
1^st^ heat 40 DIM	Cluster (0)	66	0.37	0.22	0.09	1.44	0.94	2.20
	Cluster (1)	160	Ref					
1^st^ heat 60 DIM	Cluster (0)	66	0.27	0.17	0.10	1.32	0.95	1.82
	Cluster (1)	160	Ref					
1^st^ heat 90 DIM	Cluster (0)	66	0.20	0.15	0.19	1.22	0.91	1.65
	Cluster (1)	160	Ref					
1^st^ service 60 DIM	Cluster (0)	66	0.04	0.20	0.85	1.04	0.71	1.52
	Cluster (1)	160	Ref					
1^st^ service 90 DIM	Cluster (0)	66	0.00	0.16	1.00	1.00	0.74	1.36
	Cluster (1)	160	Ref					
1^st^ service 120 DIM	Cluster (0)	66	-0.02	0.15	0.91	0.98	0.73	1.32
	Cluster (1)	160	Ref					
Pregnancy 100 DIM	Cluster (0)	66	0.24	0.26	0.34	1.28	0.77	2.12
	Cluster (1)	160	Ref					
Pregnancy 150 DIM	Cluster (0)	66	0.16	0.20	0.43	1.17	0.79	1.74
	Cluster (1)	160	Ref					
Pregnancy 200 DIM	Cluster (0)	66	0.10	0.18	0.57	1.11	0.78	1.56
	Cluster (1)	160	Ref					

DIM=Number of days in milk, HR=Hazard ratio, CI=Confidence interval.

Although not statistically significant, these findings suggest a biological association between early postpartum behavior and estrus onset. The elevated HRs in Cluster 0 point to accelerated estrus resumption, supporting the utility of behavioral monitoring in optimizing estrus detection and insemination timing.

No significant differences in other reproductive metrics were observed between clusters. However, Cluster 0 cows achieved first service slightly earlier, with a 4% higher likelihood at 60 DIM (HR = 1.04, p = 0.85). Similarly, conception rates at 100, 150, and 200 DIM were marginally higher in Cluster 0, though not statistically significant.

These trends underscore the potential influence of early behavioral patterns on reproductive outcomes. Further studies involving larger, multidimensional datasets and varying management systems are warranted to refine sensor-based estrus detection strategies.

### Milk production dynamics across parity and clusters

**Figure 5:**
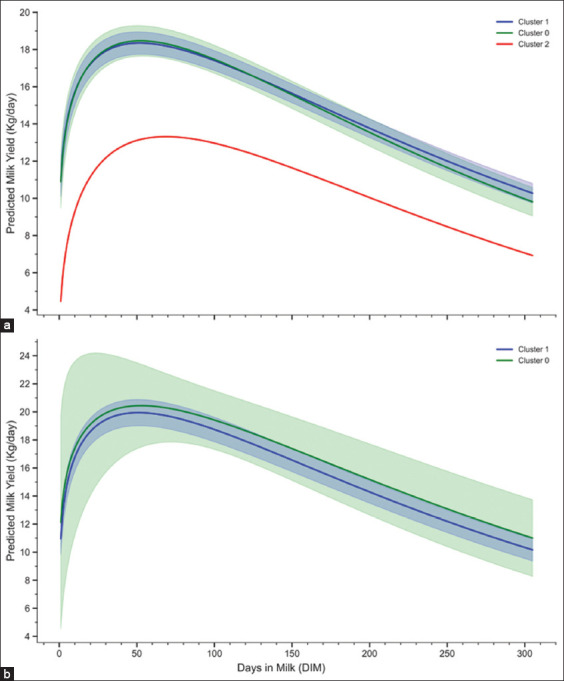
The figure demonstrates (a) predicted milk yield (kg/day) of primiparous cows (n = 173) across three cluster groups (0, 1, and 2) and (b) predicted milk yield (kg/day) of multiparous cows (n = 53) within two cluster groups (0, and 1), with 95% confidence interval.

Tables 3 and 4 and Figures 5a and b illustrate milk production dynamics by parity and behavioral cluster, modeled using the Wood gamma function [17]. Differences in peak time, peak yield, persistency, and cumulative yield were observed across clusters.

For primiparous cows (Table 3 and Figure 5a), Cluster 1 cows peaked at 52.92 ± 2.59 DIM with an average yield of 18.68 ± 0.30 kg/day. Their cumulative 305-DIM yield was 4550.29 ± 7.09 kg. Cluster 0 cows peaked earlier at 50.82 ± 3.45 DIM with a slightly higher peak yield of 18.95 ± 0.43 kg/day and a cumulative yield of 4512.76 ± 104.95 kg. The cow in Cluster 2 exhibited a delayed peak (68.02 DIM) with a low peak yield (13.31 kg/day), followed by a sharp decline – indicating possible physiological or environmental stress.

For multiparous cows (Table 4 and Figure 5b), both clusters outperformed primiparous groups. Cluster 1 peaked at 51.99 ± 2.97 DIM (20.24 ± 0.48 kg/day) with a cumulative yield of 4816.38 ± 114.27 kg at 305 DIM. Cluster 0 cows peaked slightly earlier (47.45 ± 13.91 DIM) and produced the highest overall yield (4896.57 ± 252.14 kg). Despite higher variation in Cluster 0, both clusters followed typical lactation trajectories with early peaks and gradual declines.

Multiparous cows in Clusters 0 and 1, on the other hand, reached their peak early on, at 47.45 ± 13.91 DIM and 51.39 ± 3.11 DIM, respectively, and had slightly higher peak yields of 20.92 ± 1.06 kg/day and 20.24 ± 0.48 kg/day. This pattern suggests that the lactation strategy favors an early, intense production phase, followed by a more pronounced decline. Despite the rapid decline following the peak, these animals achieved the highest cumulative yield among multiparous groups at 4896.57 ± 252.14 kg per 305 DIM, marginally surpassing Cluster 1’s output yield of 4816.38 ± 114.27 kg per 305 DIM. Although no statistically significant differences were observed between clusters, Figure 5b demonstrates higher variability in production within Cluster 0, suggesting higher individual variation in lactation efficiency and environmental adaptation. The wider CI in Figure 5b visually highlights this variability, reinforcing the importance of considering both individual-level differences and inter-individual-level variability when inspecting production trends in the herd.

These findings confirm that parity and cluster classification significantly influence lactation patterns. Multiparous cows consistently produced higher yields than primiparous cows. Although differences across clusters were not statistically significant, clustering helped uncover distinct lactation trajectories. This classification has practical value in refining feeding strategies and herd management practices tailored to specific lactation profiles.

### Validation of Wood’s lactation curve model

Validation of the Wood lactation model (Table 5) demonstrated moderate predictive performance across parity and cluster groups. R² values ranged from 0.69 to 0.83 in primiparous cows, with the lowest value observed in Cluster 2 (single cow), and from 0.80 to 0.82 in multiparous cows. Mean absolute error (MAE) ranged from 1.05 to 1.48 across all groups, indicating generally stable lactation patterns. These results suggest that the Wood model provides reliable estimates of milk yield in tropical conditions, despite minor variability across parities and clusters.

## DISCUSSION

### Behavioral dynamics in dairy cows: Temporal trends and implications

This study presents a comprehensive analysis of behavioral dynamics in Holstein Friesian cows by examining postural behavioral indicators – including rolling activity, rest time, rest per bout, and restlessness ratio – across both primiparous and multiparous cows using cluster analysis from −3 to 30 DIM. Marked behavioral distinctions emerged between parity groups, reflecting their physiological and metabolic adaptations to the demands of early lactation. These adaptations varied across clusters and parity, offering meaningful insights into herd behavior.

Primiparous cows in Clusters 0, 1, and 2 demonstrated elevated rolling activity during early lactation, with Cluster 1 peaking at approximately 300 min/day soon after calving. This heightened activity likely reflects physiological and behavioral adjustments to lactation onset [44], driven by calving-related stress [45, 46] and increased energy demands [14, 17]. Conversely, multiparous cows displayed slightly reduced activity levels compared to primiparous counterparts, suggesting more efficient physiological adjustment. All animals generally returned to baseline activity within 2 weeks postpartum, consistent with earlier findings [10]. However, further investigation is warranted to elucidate the interplay of physiological and environmental adaptation in diverse populations, contributing to improved welfare strategies.

Resting behavior, a key indicator of cow welfare and productivity, revealed parity- and cluster-specific differences. A general decline in resting time was observed shortly after calving in all groups (Figures 2a and b) [46–48]. Primiparous cows in Clusters 0 and 1 maintained consistently lower resting times compared to multiparous cows. Notably, the primiparous cow in Cluster 2 exhibited an excessive resting duration exceeding 1200 min/day, potentially signaling underlying metabolic or health challenges [49, 50]. Importantly, resting times did not return to pre-calving levels by 30 DIM, underscoring the sustained impact of calving, milking routines, and management practices [10, 44, 46, 47]. These findings corroborate previous reports parity-related resting disparities [44, 48, 51] and expand on earlier research with a longer observation window.

Analysis of rest per bout durations offered insights into recovery from parturition and management-related stress [52]. Both parity groups in Clusters 0 and 1 maintained stable, moderate rest per bout durations (~90 min/day), suggesting effective adaptation to parturition and routine milking schedules [48, 53]. However, the primiparous cow in Cluster 2 demonstrated significantly extended rest durations post-calving, likely reflecting compromised health or environmental stress exposure. Overall, primiparous cows showed more consistent bout durations compared to their multiparous counterparts (Figures 3a and b), possibly due to acclimatization to new milking protocols [44, 48]. Multiparous cows, being heavier and more physically mature, generally exhibited longer resting durations per bout due to reduced ease in transitioning between postures [44, 48, 52].

The restlessness ratio further illuminated adaptive behavioral patterns. Both primiparous and multiparous cows demonstrated heightened restlessness on the day of calving [10, 46, 47]. Primiparous cows in Cluster 1 had the highest restlessness scores, while those in Cluster 0 exhibited more moderate responses, suggesting more rapid adjustment to postpartum routines [10, 44, 48]. A similar pattern was noted in multiparous cows, likely due to physiological or metabolic constraints reducing their responsiveness to environmental shifts.

Variations in restlessness ratio corroborate findings from both temperate and tropical production systems, indicating its relevance to reproductive function. Elevated restlessness in temperate zones has been linked to delayed estrus [44, 53], whereas in tropical climates, it is often associated with environmental stressors such as heat, which impair estrus expression and insemination outcomes [54–56]. Cluster 1 cows, which exhibited higher restlessness and delayed estrus, support the hypothesis that behavioral stress impedes reproductive recovery. These observations reinforce the importance of integrating behavioral metrics into estrus detection systems, especially under tropical stress conditions.

### Reproductive performance and behavioral adaptation

This study evaluated the reproductive outcomes of Holstein Friesian cows in a tropical climate, emphasizing the influence of behavioral patterns and lactation dynamics. Cluster 1 cows exhibited increased restlessness, shorter rest durations, and altered activity patterns – factors likely contributing to delayed estrus and prolonged time to first service compared to Cluster 0. While statistical significance was not reached, these trends align with previous evidence suggesting that reduced rest and heightened restlessness may hinder reproductive recovery [53, 54].

Cows in Cluster 0 demonstrated a 44% greater likelihood of resuming estrus by 40 DIM (HR = 1.44, p = 0.09), a trend that strengthened over time. Their lower restlessness and higher rest times suggest reduced physiological stress and more efficient estrus manifestation [57–59]. Moreover, stable lactation patterns in Cluster 0 indicate lower early lactation stress, enabling greater energy allocation toward reproductive restoration [50, 60]. These observations align with prior research showing that lower metabolic strain and smoother LCs promote faster reproductive recovery in tropical settings [55, 56].

Cluster 0 cows also experienced shorter intervals to first service (e.g., HR = 1.04, p = 0.85 at 60 DIM), further supporting the notion that reduced behavioral stress facilitates postpartum recovery. Their longer rest periods and lower restlessness ratios likely promoted uterine involution and ovarian cyclicity [61, 62], optimizing reproductive readiness [49, 50].

Although conception rates between clusters did not differ significantly, Cluster 0 cows demonstrated higher success rates – 28% greater at 100 DIM (HR = 1.28, p = 0.34), with continued improvement through 200 DIM. These patterns suggest that behavioral stability and lower lactation stress promote favorable reproductive conditions, particularly under tropical stressors known to compromise estrus and oocyte quality [55, 61, 63].

Tailored management interventions that reduce restlessness, promote rest, and support balanced lactation profiles can substantially enhance postpartum recovery and fertility in tropical dairy herds.

### Milk production dynamics and lactation modeling

The clustering analysis revealed substantial distinctions in milk production dynamics across parity and behavioral cluster groups, emphasizing the value of behavioral metrics in shaping lactation trajectories. Differences were evident in peak time, yield, persistency, and cumulative milk production (Tables 3 and 4, Figures 5a and b). The Wood gamma function model [17] effectively captured lactation variability when combined with early behavioral metrics.

Among primiparous cows, Cluster 1 achieved greater persistency and cumulative yield compared to Cluster 0, despite a slightly delayed peak (Table 3 and Figure 5a). This suggests that elevated activity and restlessness in Cluster 1 (Figures 1a and 4a) did not adversely impact sustained milk output. These findings are consistent with previous studies by Josefson *et al*. [64] and Marumo *et al*. [65], indicating that primiparous cows often compensate for early stress through enhanced persistency.

The single cow in Cluster 2 exhibited an atypical LC, with a delayed peak and lower yield (Table 3 and Figure 5a), alongside behavioral anomalies – such as excessive rest time (>1200 min) and rest per bout (>700 min) (Figures 2a and 3a). These abnormalities may indicate health issues or negative energy balance [14, 50, 66] and support previous findings by Lee *et al*. [14], Cattaneo *et al*. [50], and Singh and Bhakat [68] linking atypical LCs with metabolic stress, suboptimal body condition recovery, and heightened disease risk. Future studies should refine clustering models to better identify LC deviations and assess their predictive value for health and productivity.

In multiparous cows, Cluster 0 demonstrated earlier peak times and greater persistency than Cluster 1 (Table 4 and Figure 5b). The sharper post-peak decline in Cluster 1 may reflect physiological strain and elevated behavioral stress during early lactation. Increased activity and restlessness, along with reduced rest durations (Figures 1b and 4b), suggest a maladaptive response to metabolic and environmental demands [53, 69, 70].

Cows in Cluster 0 of both parity groups exhibited more consistent behavioral and lactation profiles. Reduced agitation, extended resting durations, and stable rest per bout metrics facilitated recovery from parturition stress and supported sustained lactation performance. These findings align with earlier reports indicating higher yield and persistency in multiparous cows, particularly with stable behavioral patterns [14, 71, 72]. Conversely, higher persistency among primiparous cows, despite lower peak yields, supports previous claims regarding their physiological immaturity and limited mammary gland development [44, 65, 73, 74].

Behavioral traits, particularly rest duration, appear strongly associated with milk yield. Previous studies by Tucker *et al*. [53] and McWilliams *et al*. [75] suggest milk output can increase by 1.36–2.72 kg/day through optimized early rest periods. In this study, cows in Cluster 0 experienced fewer behavioral fluctuations and maintained higher production consistency during early lactation.

### LC modeling and limitations

The Wood gamma function model [17] successfully captured LC characteristics across clusters and parities. In primiparous Clusters 0 and 1, the model effectively reflected gradual peak onset and high persistency. Cluster 0 cows in the ≥2 parity group achieved the highest cumulative yield at 305 DIM. Their flatter LC trajectory is characteristic of younger cows still undergoing mammary gland development [73, 74].

Among multiparous cows, the model fit was equally strong, capturing more intense production early on and subsequent yield decline. For Cluster 0, early peak and high cumulative output were followed by a steep drop, consistent with previous reports by Lee *et al*. [14] and Masía *et al*. [76] linking high early yield to accelerated metabolic decline.

These results highlight contrasting lactation strategies: Multiparous cows achieve higher yield but exhibit sharper declines, whereas primiparous cows sustain lower but more persistent production. Accurate modeling of both typical and atypical LCs requires high-resolution tools capable of distinguishing nuanced curve profiles.

Notably, this study faced several limitations. Sensor placement on the right hind leg may introduce inconsistencies due to variation in movement or positioning. Cluster 2’s sample size (n = 1) limited statistical power for reproductive comparisons. In addition, the single-farm setting and use of one sensor model constrain generalizability. Future research should incorporate multisensor approaches, validate findings across diverse environments, and assess cross-farm reproducibility to enhance external validity and applicability.

## CONCLUSION

This study provides an integrative assessment of early postpartum behavioral dynamics and their association with reproductive performance and milk production in Holstein Friesian cows under tropical conditions. By employing K-means clustering on sensor-derived postural behavioral variables – namely, rolling activity, rest time, rest per bout, and restlessness ratio – the study identified distinct behavioral phenotypes across parity groups. The application of the Wood gamma function model [17] further enabled the characterization of LCs, elucidating variations in peak yield, persistency, and cumulative milk production across clusters.

The results demonstrated that cows in Cluster 0, characterized by more stable activity levels, longer rest durations, and lower restlessness ratios, exhibited superior reproductive performance trends and higher cumulative milk yields. In contrast, cows in Cluster 1, which displayed elevated early postpartum activity and restlessness, experienced delayed estrus resumption and a steeper decline in milk yield post-peak. The single animal in Cluster 2 exhibited atypical behavioral and lactation profiles, indicating possible underlying health or metabolic disorders. These findings underscore the relevance of behavioral clustering in understanding the physiological responses of dairy cows during early lactation, particularly in high-stress tropical environments.

A key strength of this study lies in its integration of high-resolution behavioral sensor data with reproductive and lactation outcomes over an extended postpartum window (−3 to 30 DIM), combined with rigorous statistical modeling using Cox regression and LC fitting. The use of cluster-based behavioral profiles provided a novel framework for identifying animals at risk of reproductive inefficiency or suboptimal milk production, offering actionable insights for precision dairy management.

However, several limitations warrant consider-ation. First, the use of a single commercial dairy farm and a limited number of animals in certain clusters (particularly Cluster 2) restricts the generalizability and statistical power of the findings. Second, reliance on a single sensor placement (right hind leg) may introduce measurement variability due to differences in locomotion and posture. Third, the Wood model, while effective in capturing general lactation trends, does not account for external modifiers such as disease, heat stress, or nutritional changes.

Future studies should address these limitations by incorporating larger, multi-farm datasets with diverse environmental and managerial conditions. Integration of multi-sensor platforms, real-time data streaming, and advanced machine learning techniques may further enhance the predictive capacity of behavioral models. Moreover, exploring the causal mechanisms linking behavioral traits to reproductive physiology and metabolic status could refine early detection systems for improving fertility and productivity in tropical dairy systems.

In summary, this study highlights the practical utility of sensor-based behavioral clustering in identifying lactation and reproductive patterns and advocates for the development of precision tools tailored to the environmental and physiological realities of tropical dairy farming.

## AUTHORS’ CONTRIBUTIONS

AR and CI: Conceptualization, methodology development, investigation, data acquisition, and drafted and edited the manuscript. KA: Data acquisition and drafted, reviewed, and edited the manuscript. TS: Interpretation of data and reviewed and edited the manuscript. HH: Interpretation of data, supervised the study, and reviewed and edited the manuscript. All authors have read and approved the final version of the manuscript.
